# Application of the Intermittency Ratio Metric for the Classification of Urban Sites Based on Road Traffic Noise Events

**DOI:** 10.3390/s19235136

**Published:** 2019-11-23

**Authors:** Giovanni Brambilla, Chiara Confalonieri, Roberto Benocci

**Affiliations:** 1 CNR-INM Dept. Acoustics and Sensors “O.M. Corbino”, via del Fosso del Cavaliere 100, 00133 Rome, Italy; giovanni.brambilla@artov.inm.cnr.it; 2 Department of Earth and Environmental Sciences (DISAT), University of Milano-Bicocca, Piazza della Scienza 1, 20126 Milano, Italy; c.confalonieri12@campus.unimib.it

**Keywords:** road traffic noise, noise events, intermittency ratio, urban sites classification

## Abstract

Human hearing adapts to steady signals, but remains very sensitive to fluctuations as well as to prominent, salient noise events. The higher these fluctuations are, the more annoying a sound is possibly perceived. To quantify these fluctuations, descriptors have been proposed in the literature and, among these, the intermittency ratio (*IR*) has been formulated to quantify the eventfulness of an exposure from transportation noise. This paper deals with the application of *IR* to urban road traffic noise data, collected in terms of 1 s A-weighted sound pressure level (SPL), without being attended, monitored continuously for 24 h in 90 sites in the city of Milan. *IR* was computed on each hourly data of the 251 time series available (lasting 24 h each), including different types of roads, from motorways to local roads with low traffic flow. The obtained hourly *IR* values have been processed by clustering methods to extract the most significant temporal pattern features of *IR* in order to figure out a criterion to classify the urban sites taking into account road traffic noise events, which potentially increase annoyance. Two clusters have been obtained and a “non-acoustic” parameter *x*, determined by combination of the traffic flow rate in three hourly intervals, has allowed to associate each site with the cluster membership. The described methodology could be fruitfully applied on road traffic noise data in other cities. Moreover, to have a more detailed characterization of noise exposure, *IR*, describing SPL short-term temporal variations, has proved to be a useful supplementary metric accompanying *L_Aeq_*, which is limited to measure the energy content of the noise exposure.

## 1. Introduction

Noise pollution has been estimated as the second major environmental health risk after air pollution in Europe [1]. The noise health effects may emerge directly via autonomous stress reactions to the physical exposure or indirectly via negative affective states, for example the evoked annoyance. Noise annoyance may interfere with daily activities, rest or sleep, and can be accompanied by negative emotional and behavioral responses such as anger, displeasure, exhaustion and by stress-related symptoms [2,3,4].

There is clear evidence in the literature that annoyance and sleep effects depend not only on sound energy, described by metrics like *L_Aeq_*, but also by the characteristics of noise events, which can be quantified by different metrics proposed in the literature, as those reviewed in [5]. It is well known that human hearing is able to adapt to steady noise easier than to the sound pressure level (SPL) fluctuations, as well as to prominent, salient noise events [6,7]. The higher these fluctuations are, the more annoying a sound is possibly perceived. Road traffic noise is typically characterized by the noise events due to the single vehicle pass-by, where the temporal structure of SPL varies between local one-lane city roads, showing highly intermittent noise, up to wide multi-lane motorways, producing a nearly continuous noise with very limited SPL fluctuations. To quantify these SPL fluctuations, common approaches either apply thresholds to detect events exceeding such thresholds and count number and duration of these events, or use SPL statistics, like percentile levels *L_A1_*, *L_A5_* and *L_A10_*, namely the A-weighted SPL exceeded for 1%, 5% and 10% of the measurement time, respectively.

Recently, a new descriptor has been proposed [8], describing the eventfulness (or intermittency) of transportation noise exposure, taking into account both number and magnitude of noise events during a certain time period. The metric, named intermittency ratio (*IR*) and introduced within the framework of the SiRENE project, can be derived either directly from acoustic measurements or calculated from traffic and geometric data for any transportation noise source and any time period. A recent survey, performed on a stratified random sample of 5592 residents exposed to transportation noise all over Switzerland, has shown that for road traffic noise *IR* has an additional effect on the percentage of highly annoyed people and can explain shifts of the exposure-response curve of up to about 6 dB between low *IR* and high *IR* exposure situations, possibly due to the effect of different durations of noise-free intervals between events [9]. Moreover, a parameter study, based on calculations, has showed the dependency of *IR* on source–receiver distance, traffic volume, the percentage of heavy vehicles and travelling speed [10].

The metric *IR* has been determined on the 1 s A-weighted SPL from road traffic, without being attended, monitored continuously for 24 h in 90 sites in the city of Milan. It was computed on each hourly data of the 251 time series available (lasting 24 h each), including different types of roads, from motorways to local roads with low traffic flow. The obtained hourly *IR* values have been processed by clustering methods to extract the most significant temporal pattern features of *IR*, in order to figure out a criterion to classify the urban sites considering road traffic noise events, which potentially increase annoyance. Two clusters have been determined and a “non-acoustic” parameter *x*, calculated by combination of the traffic flow rate in three hourly intervals, has allowed us to associate each site with the cluster membership. Furthermore, binomial logistic regression has been applied to develop a model to predict the cluster membership on the basis of the *IR* time patterns. The performance of the model, determined comparing the predicted classification of the test data subset with that obtained by the cluster analysis, was satisfactory.

## 2. Materials and Methods

### 2.1. Acoustic Data Set

In the framework of the LIFE DYNAMAP project, a large road traffic noise monitoring survey was carried out in the entire area of Milan to collect a database containing noise data related to the city road network [11]. From this database a set of 90 sites have been considered to represent the different types of roads according to the Italian functional road classification, that is motorway (class “A”), thoroughfare roads (class “D”), urban district roads (class “E”) and urban local roads (“F”). The distribution of the sites among these classes is given in Table 1, together with the number of 24-hour time series of 1 s A-weighted sound pressure level (SPL) from road traffic monitored continuously by a class 1 sound level meter with the microphone placed at 4 m above the road. In some sites the unattended monitoring has been performed on more consecutive days. The monitoring has been performed on weekdays only (Monday to Friday), without rain and with wind speed less than 5 m/s. Noise events not associated with road traffic have been visually detected and manually masked before further data processing. The microphone was placed close to the road to reduce the influence of noise from other sources. Thus, the noise data are not representative of the real exposure of residents, living at greater distances from the road, especially where the road has a multi-lane geometrical configuration and even further away if the lanes are separated by a median strip area. In particular, the inhabitants’ exposure is most likely overestimated because the values of *IR* and *L_Aeq_* are greater than those at the road facing building façades (i.e., for *IR* dependency on source–receiver distance see Figure 4 in [10]). Details on the numbers of lanes for each direction and the average distance of the microphone from the roadside are given in Table 1.

### 2.2. Intermittency Ratio IR Formulation

A noise event can be characterized by its maximum level, its sound exposure level (SEL), its “emergence” from background noise, its duration, or by the slope of rise of the level. For the characterization of the “eventfulness” of a noise exposure, the event continuous equivalent level *L_eq,T,Events_* is introduced in the *IR* formulation, which accounts for all sound energy contributions that exceed a given threshold, that is clearly stand out from background noise. This parameter is referred to the overall continuous equivalent level *L_eq,T,tot_* for the measurement time *T* to give the following formulation of *IR* [8]:

(1) $$IR\;=\frac{10^{0.1L_{eq,T,Events}}}{10^{0.1L_{eq,T,tot}}}\;\cdot 100\;\left[\%\right].$$

**Figure 1:**
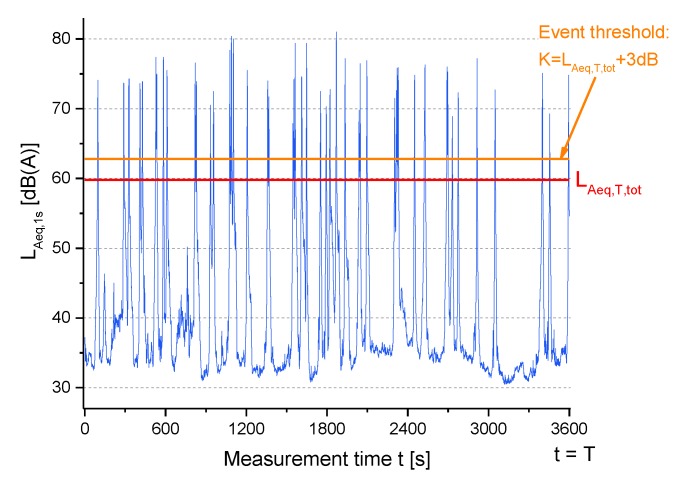
A-weighted sound pressure level versus time *t* (*T* = 1 h) for an urban local road. The sound pressure levels (SPLs) above the threshold *K* are events contributing to determine *L_eq,T,Events_*. For the plotted hourly SPL time history intermittency ratio (*IR*) = 93.3%, *L_Aeq,T,tot_* = 59.8 dB(A), number of events above *K* threshold = 45 and *L_Aeq,T,Events_* = 59.5 dB(A).

A single pass-by only contributes to *L_eq,T,Events_* if its SPL exceeds a given threshold *K* determined by:

(2) $$K\;=L_{eq,T,tot}+C\;\left[\mathrm{dB}\right],$$

where *C* might be between 0 and 10 dB. For low values of *C*, almost any situation produces a high *IR*, whereas high values of *C* almost always produce low *IR*. The balance between these extreme cases was investigated by numerical simulations of various traffic situations and resulted in *C* = 3 dB [8]. This value has not been set based on any verified psychoacoustic principle, but was derived empirically. As pointed out in [8], “The question of how much an event really has to stand out from background noise in order to be termed “event” by normal listeners depends on various other parameters”, like the attentional, cognitive and emotional situation of the listener [6]. By definition, *IR* only takes values between 0% and 100%. An *IR* > 50% means that more than half of the sound exposure is caused by “distinct” pass-by events. In situations with only events that clearly emerge from background noise (e.g., a receiver very close by a road), *IR* yields values close to 100%. For example, Figure 1 shows the A-weighted SPL time history (measurement time *T* = 1 h) for an urban local road together with the corresponding *L_Aeq,T,tot_* and the threshold *K* used to detect the events (all the SPLs above *K*), which determine *L_Aeq,T,Events_*.

### 2.3. Data Processing and Analysis

A script running in the “R” environment, version 3.5.1 [12], has been written to import each of the 24-h time series as input in terms of text file (four columns with date, time, SPL in dB(A) at 1 s intervals and a code to indicate the corresponding source, which is road traffic noise or something else). The reference measurement time *T* was chosen equal to 1 h, as this time frame is established by the Italian legislation for road traffic noise measurement. Besides this requirement, the chosen measurement time *T* of 1 h was considered a reasonable compromise between longer time (i.e., 24 h, day and night periods, etc.) and shorter ones (i.e., 30 min or even shorter). For each *T* of 1 h, the output data were exported to an Excel file, including:

- The overall *L_Aeq_* hourly value (dB(A));
- The hourly value of intermittency ratio *IR* (%) and the corresponding number of events;
- For each detected noise event the corresponding start time, the duration (s) and the sound exposure level (SEL; dB(A)).

In addition for each site the hourly traffic flow was provided by the Municipal Agency of Mobility, Environment and Land of Milan (AMAT). The data were calculated by a model of traffic applied to the city road network.

The statistical analysis of the collected data was carried out by the software “R” [12]. For the sites where the noise monitoring lasted more days the median value of *IR* for each hour was determined, as this parameter is less influenced by the presence of outliers. Thus a matrix of 90 (sites) × 24 (hours) = 2160 values of hourly *IR* was used as input of the subsequent cluster analysis performed to find out the similarities in the *IR* time patterns.

To fulfill such an objective, hierarchical clustering, an unsupervised machine learning method for data classification, was applied. This method does not require to pre-specify the number of clusters to be generated and the output is a tree-based representation of the observations (dendrogram) showing the sequence of cluster formation and the distance at which each fusion takes place. Previously, for each hour the *IR* values have been scaled (mean = 0 and standard deviation = 1). The Euclidean distance has been considered to represent the similarity between pairs of observations. Complete-linkage clustering was considered: at the beginning of the process, each element is in a cluster of its own and, afterwards, the clusters are sequentially combined into larger clusters until all elements end up being in the same cluster. Different clustering methods available in the “clValid” R package, version 0.6-6 [13], were applied. In particular, six methods were considered, that is hierarchical, partitioning around medoids (PAM), k-means, divisive analysis clustering (DIANA), model-base clustering and self-organizing tree algorithm (SOTA). For the sake of simplicity, minimal discrimination was considered, that is two clusters for both the sites and the hourly time intervals. The clustering performance of the methods was ranked according to seven parameters, namely connectivity, silhouette width and Dunn index (combining measures of compactness and separation of the clusters), the average proportion of non-overlap (APN), the average distance (AD), the average distance between means (ADM) and the figure of merit (FOM). The method selected as “optimal” on the basis of the above parameters was applied to obtain two clusters of *IR* patterns for both the sites and the hourly time intervals.

Afterwards, a model was developed to predict the cluster membership on the basis of the *IR* time patterns. For this purpose the “caret” R package, acronym for “Classification And REgression Training” [14], was used. The dataset needed to be randomly divided into two subsets, one for training the model and the other to test it and evaluate its classification performance. The binomial logistic regression was applied to develop the model because the dependent variable (cluster membership) was categorical with two categories. The classification performance of the model was determined comparing the predicted classification of the test data subset with that obtained by the cluster analysis.

## 3. Results

**Figure 2:**
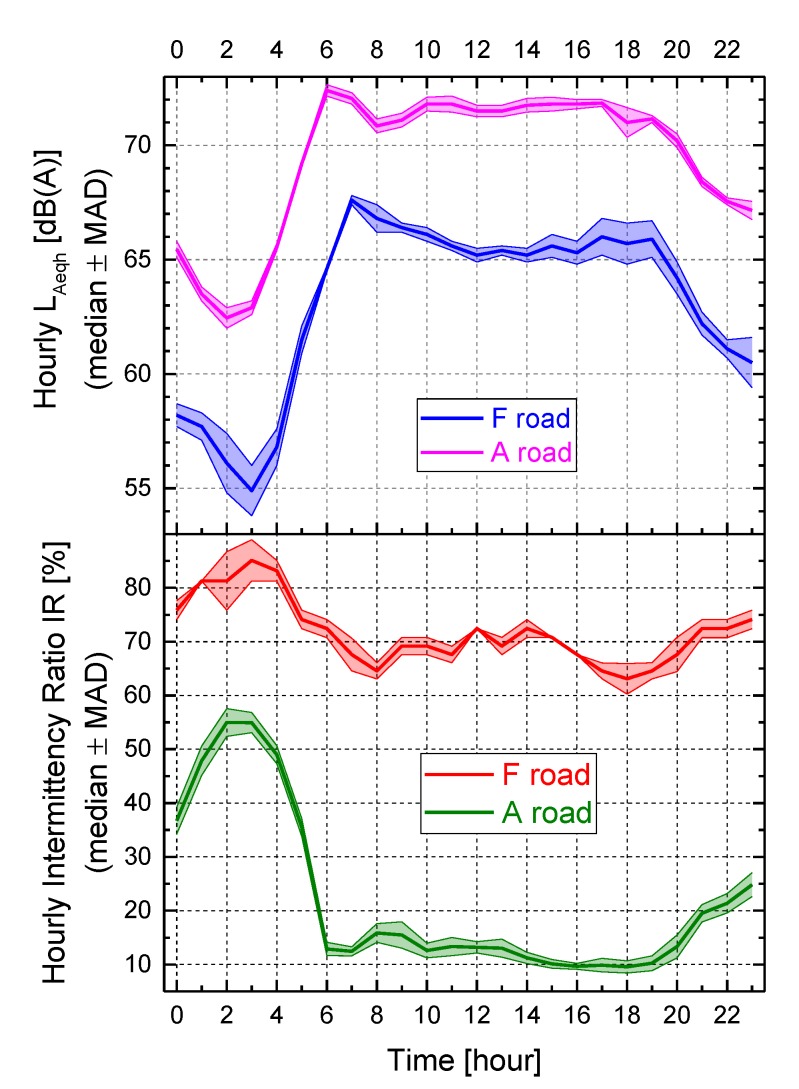
Example of the obtained 24-h pattern of hourly values of *L_Aeq_* and corresponding *IR* for two different types of roads, namely a motorway (class “A”) and a local street (class “F”).

Figure 2 shows an example of the obtained 24-h pattern of hourly values of *L_Aeq_* and corresponding *IR* for two different types of roads, namely a motorway (class “A”) and a local street (class “F”). The plot reports the median of the hourly values ± the median absolute deviation (MAD) because the monitoring included more than one day, namely 12 days for road “A” and 9 days for road “F”. It can be seen that road “A” was always much noisier than road “F” (hourly *L_Aeq_* average differences across the hours of about 6 dB) and shows always lower *IR* values than road “F” (hourly *IR* average difference across the hours of about −50%, and less pronounced (−30%) during the night). The lower *IR* values observed for road “A” were due to the high traffic flow rate and speed on the motorway, resulting in a high background SPL above which the noise events did not stand out too much. This feature was clearly present in the day period from 6 to 18 h, whereas for the period from 2 to 4 h the highest values of *IR* were observed, when the reduced traffic flow allowed the increase of speed and more prominent noise events occurred above the lower background level. Road “F” shows the same behavior in the night, whereas the lowest *IR* values occurred at the traffic peak hours (8 and 18 h), when the traffic flow was highest and the increased background SPL reduced the prominence of noise events. Thus, given the very different temporal patterns of urban road traffic noise, from relative continuity to high intermittency, it would be worth to consider the *IR* metric as a supplementary quantity to *L_Aeq_*.

**Figure 3:**
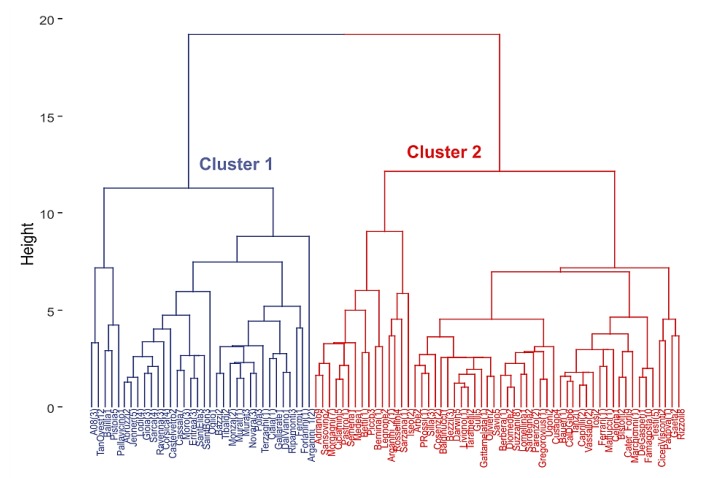
Dendrogram of the scaled *IR* hourly values obtained for the 24-h road traffic noise data monitored in the 90 sites in Milan.

Regarding clustering, the DIANA method was selected as the “optimal” clustering algorithm to divide the data set into two clusters of *IR* patterns for both the sites and the hourly time intervals. The dendrogram of the scaled *IR* hourly values obtained for the sites (matrix with rows = sites and columns = hours) is given in Figure 3. Table 2 reports the distribution of the sites across the road type and clusters. Cluster 2, on the right hand side in Figure 3, includes the majority of all the road types, whereas Cluster 1, on the left hand side in Figure 3, includes the remaining roads and all those in class “A”.

**Figure 4:**
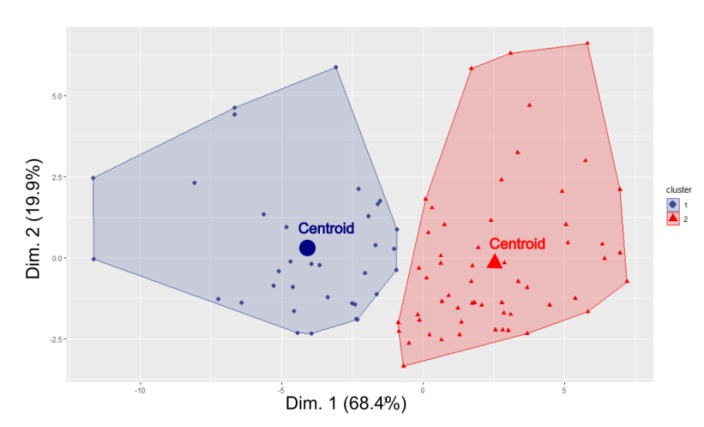
Bi-dimensional plot of the two clusters obtained by multidimensional scaling (MDS). Dimension 1 and 2 explain 68.4% and 19.9% of the variance, respectively.

The multidimensional scaling (MDS) applied to the data provided the bi-dimensional plot given in Figure 4, where the two clusters appeared satisfactorily separated and the variance explained by the two dimensions was 88.3%.

**Figure 5:**
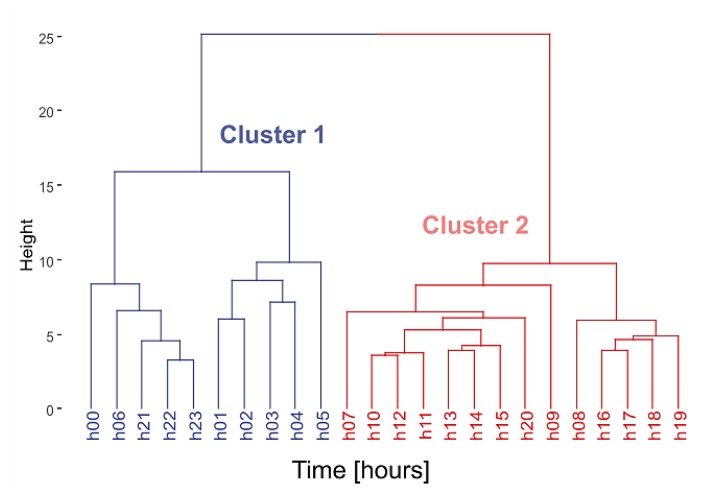
Dendrogram of the scaled *IR* hourly values as a function of the hours.

**Figure 6:**
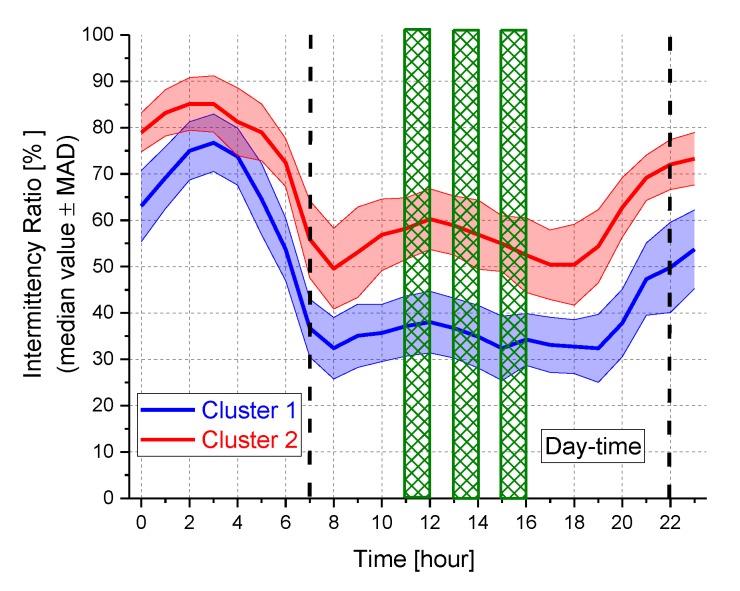
*IR* time pattern for each cluster. Green rectangles correspond to the hourly intervals showing the biggest differences between the two *IR* time patterns.

The dendrogram in Figure 5 shows the clustering in terms of hourly intervals, obtained after the transposition of the matrix containing the 2160 values of hourly *IR* (rows = hours and columns = sites). The night period (from 22 to 7 h) was clearly separated from the day-time. Regarding the *IR* time pattern for each cluster, Figure 6 reports the hourly median *IR* values ± the median absolute deviation (MAD) and the three hourly intervals showing the biggest differences between the two clusters (green rectangles). In the night period the *IR* values were the highest for both clusters because of the presence of noise events clearly emerging above the background noise. In this time period there was an overlapping between *IR* values corresponding to the two clusters. Similar median *IR* time patterns were also observed from 7 to 24 h, with Cluster 1 having lower *IR* values. As expected the night period was the most critical due to prominent noise events, which could produce an increasing of annoyance, considering also the affected activities (mainly sleep).

**Figure 7:**
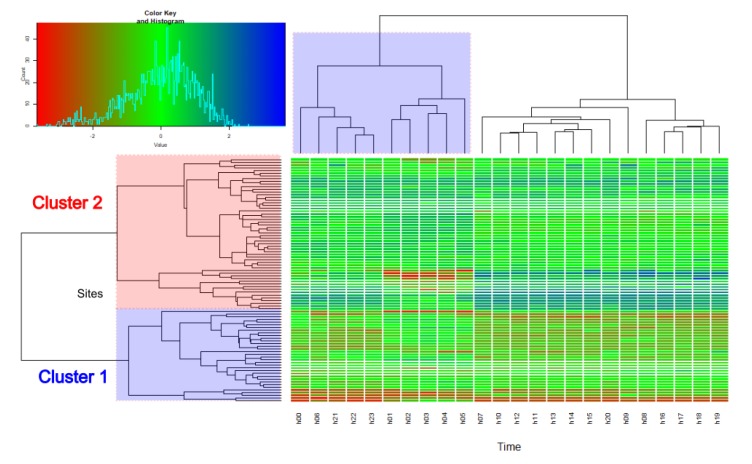
Cluster heatmap of the scaled hourly values of *IR*. On the *y* axis the sites divided into Cluster 1 (blue rectangle) and 2 (red rectangle). On the *x* axis the clustering across the hourly intervals with the night period, from 22 to 7 h, in the blue rectangle.

The above results of clustering were also plotted in terms of a heatmap, reported in Figure 7, a rectangular tiling of the data matrix with cluster trees appended to its margins, where the rows and columns of the matrix are ordered to highlight patterns [15]. The color key legend on the top left in the figure shows also the distribution of the 2160 hourly *IR* values.

The obtained *IR* time pattern for each cluster cannot be applied in a straightforward way without any linking to a specific feature of either the road or the corresponding traffic flow. As shown in Table 2, the road type was useless because each cluster included different road types. Thus, to find a “non-acoustic” parameter suitable to predict the cluster membership, the Mann-Whitney U test was performed on the hourly *IR* values to detect the hourly intervals where their differences between the two clusters were biggest. The rank descending order of these differences showed that they corresponded to the hourly intervals 15–16 h, 13–14 h and 11–12 h (see Figure 7). Thus, the traffic flows *F* in these three hours were combined according to the following relationship, similar to that previously proposed in [16]:

(3) $$x\;=\sqrt{{\left[lg\left(F_{15-16}\right)\right]}^{2}+{\left[lg\left(F_{13-14}\right)\right]}^{2}+{\left[lg\left(F_{11-12}\right)\right]}^{2}}.$$

Having a separation of the sites into two clusters, binomial logistic regression was applied to develop a model to predict this classification. This is a statistical model that in its basic form uses a logistic function (known as “S” shape or sigmoid curve) to model a binary dependent variable, having only two possible values. In such a model, the cluster membership was considered as a dependent variable, in particular Cluster 1 was labeled “0” and Cluster 2 was labeled “1”, and the “non-acoustic” parameter *x* was taken as an independent variable (predictor). The split ratio = 0.7 was used for randomly sub-setting the data set for training the classification model (63 sites) and, afterwards, to test it (27 sites). At the end of the training process, the model equations in terms of probability *P* of an observation to belong to Cluster 2 (Y = 1) was obtained as follows:

(4) $$P\left(Y=1\right)=\frac{1}{1+e^{\left(-6.84+1.26x\right)}}.$$

**Figure 8:**
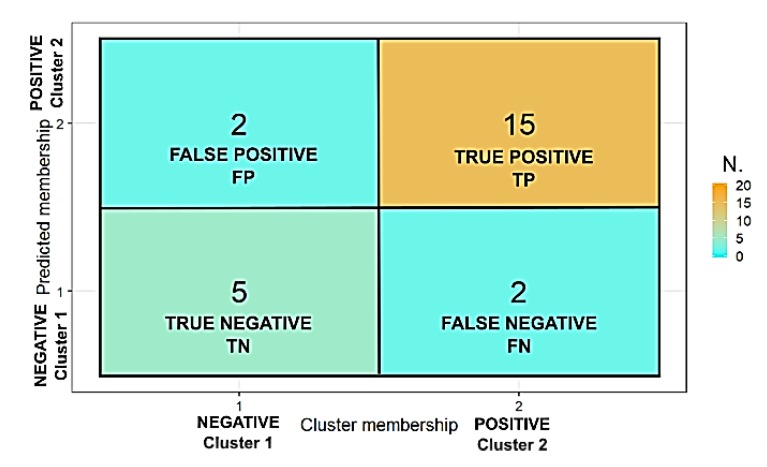
Confusion matrix of the classification model applied to the test dataset.

**Figure 9:**
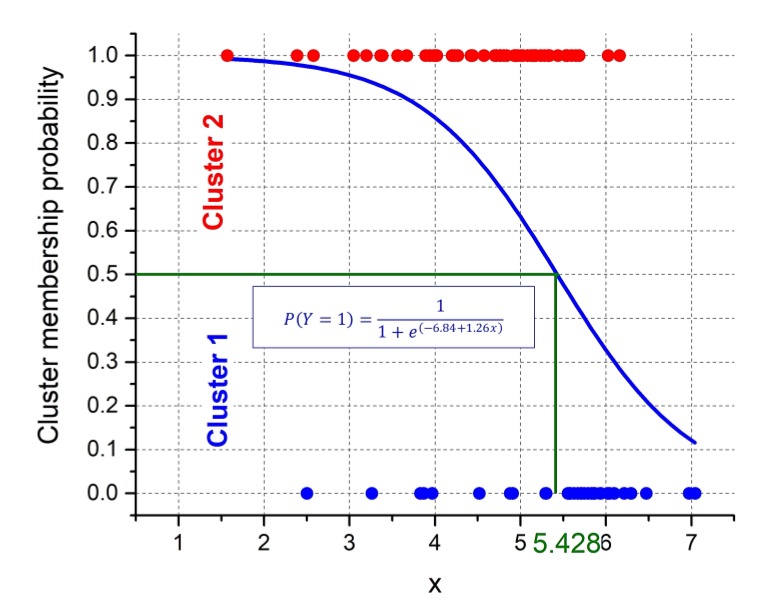
Cluster membership (blue dots = Cluster 1 and red dots = Cluster 2) obtained by the divisive analysis (DIANA) clustering compared with the probabilities predicted by the logistic regression (blue curve obtained by Equation (4)). Probabilities *p* ≤ 0.5 and p > 0.5 correspond to Cluster 1 and 2, respectively. The threshold for the “non-acoustic” parameter x to discriminate between the cluster membership is reported in green.

The classification model was applied to the test dataset in order to evaluate its classification performance and the obtained confusion matrix, a table counting how often each combination of known categories (the clusters) occurred in combination with each prediction type, is reported in Figure 8. The results were satisfactory, being the model accuracy (fraction of correct predictions) equaled to 0.83, the precision (the ratio of true positives to predicted positives) and recall (the ratio of true positives over all positives) equaled to 0.88 and the Cohen’s kappa |ê = 0.60 (moderate agreement). Table 3 reports additional performance parameters. Figure 9 shows the comparison between the cluster membership (blue dots = Cluster 1 and red dots = Cluster 2) obtained by the DIANA clustering and the probabilities predicted by the logistic regression (blue curve obtained by Equation (4)). The proportion of correctly classified observations by the model was equal to 0.74.

**Figure 10:**
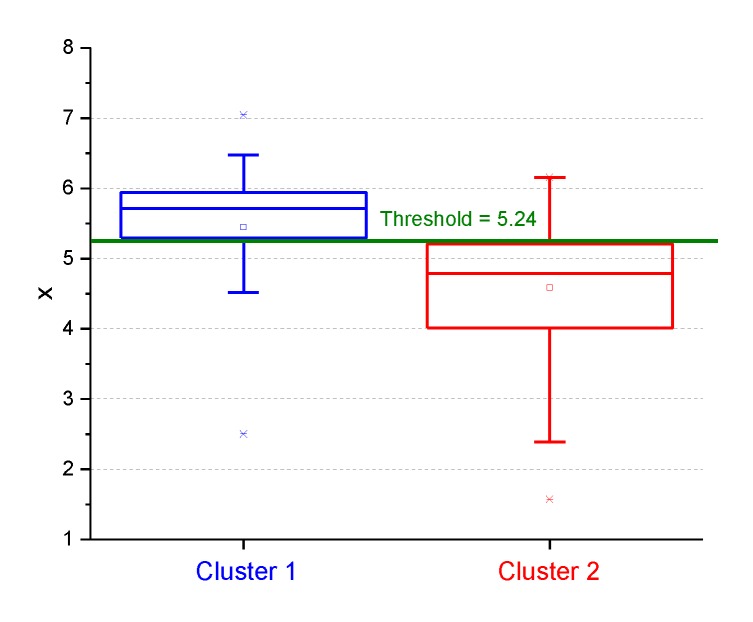
Empirical threshold value of the “non-acoustic” parameter *x* obtained for the discrimination between the two clusters (*x* = 5.24).

Regarding the effective application of the above two clusters, it is essential to determine a threshold for the “non-acoustic” parameter *x* able to discriminate between the cluster membership. Such a threshold (*x* = 5.24) was empirically determined as shown in the box plot of the *x* values reported according to the cluster membership of sites (Figure 10). This value was comparable with that obtained from the intersection of the logistic model curve with the cluster membership probability value of 0.5, shown in Figure 9 (*x* = 5.428).

## 4. Discussion

**Figure 11:**
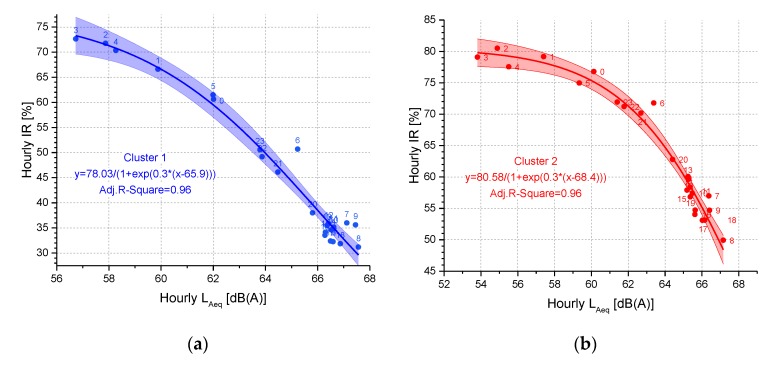
Logistic fitting of the hourly values of *IR* and *L_Aeq_* for the centroids of cluster 1 (**a**) and 2 (**b**). The area around the regression line represents the confidence bands at a 95% confidence level. The symbol labels represent the hourly intervals.

It has to be pointed out that the *IR* values calculated from the noise data provided by the noise monitoring network in Milan have some drawbacks due to some factors, like the different distance microphone-longitudinal axis of the road, the microphone proximity to the road and not where the residents live and so forth. In addition, the results of the clustering and classification model were strongly dependent on the local situation and could not be generalized to other contexts. Besides these limitations, the methodology applied could be fruitful applied in other cities and some general considerations could be drawn. For instance, the hourly *IR* and *L_Aeq_* time patterns, shown by the example in Figure 2, highlight the complementarity of these two metrics, the former describing SPL short-term temporal variation, the latter measuring the energy content of the noise exposure. In particular, for the available experimental dataset, Figure 11 reports the logistic fitting of the hourly values of these two descriptors for the centroids of cluster 1 and 2.

Due to its definition, the *IR* value ranges between the following two opposite sonic environments:

1. Sound events with low energy, not so much “emerging” from high background SPL, corresponding to a low value of *IR*;
2. Sound events with high energy, clearly “predominating” above low background SPL, corresponding to a high value of *IR*.

The sonic environment (1) occurs usually at roads with high traffic road rate, such as motorways and thoroughfare roads (road classes “A” and “D”) especially during the day-time, whereas the sonic environment (2) is usually observed at roads either with low traffic road rate, such as local roads (road class “F”) during the day-time or during the night for all the roads with the exception of motorways.

**Figure 12:**
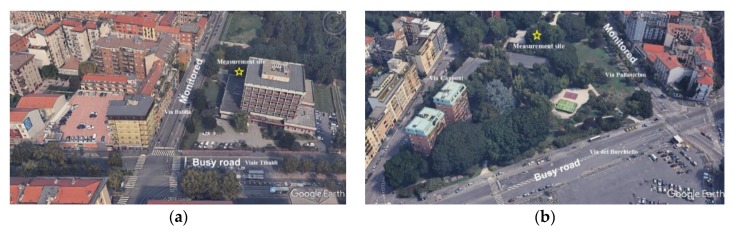
Examples of two local roads (**a** and **b**) monitored nearby a busy street (adapted from Google Earth images).

However, there might be particular cases, indeed very frequent in the urban context, where the local road is very close to a busy street whose noise is clearly influencing the sonic environment in the local road itself. In these circumstances, the low energy noise events, produced by small number of vehicle pass-by at low speed, do not emerge so much above the high background SPL produced by the nearby busy road. In the data set herewith considered there were a few sites with this feature, like the two ones shown in Figure 12. The *IR* time pattern in these sites is similar to those observed for thoroughfare roads. This is, most likely, the reason why a marginal percentage (23.1%) of local roads (class “F”) have not been grouped in the cluster containing busy roads. Thus, in the selection of sites to be monitored it is important to avoid, as much as possible, this situation, which, nevertheless, is often present in urban road network.

**Figure 13:**
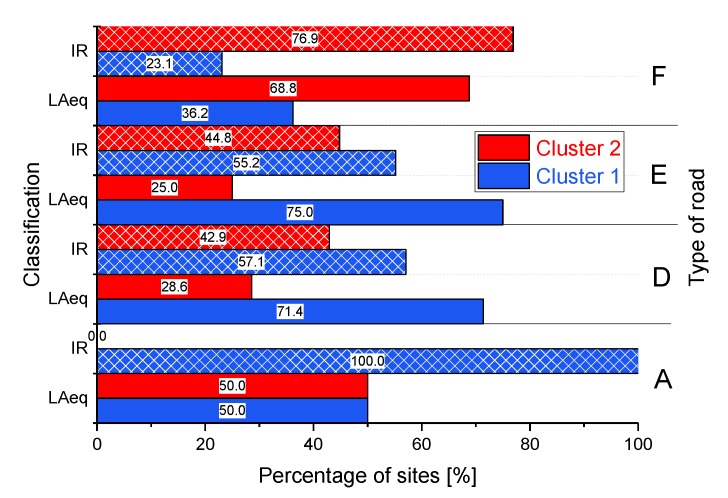
Comparison between the classifications based on *IR* and *L_Aeq_* hourly time patterns for the type of roads.

The above remarks should not be considered a weakness of the *IR* metric, but rather a reliable representation of the time pattern of the sonic environment and of the potential annoyance it might evoke. In addition, a comparison has been performed between the classification based on *IR* hourly time patterns and that provided by hourly *L_Aeq_* time patterns, the latter obtained according to the procedure detailed in [17,18]. The two classifications, as shown in Figure 13, are somewhat different, as they overlap for 64% only.

**Figure 14:**
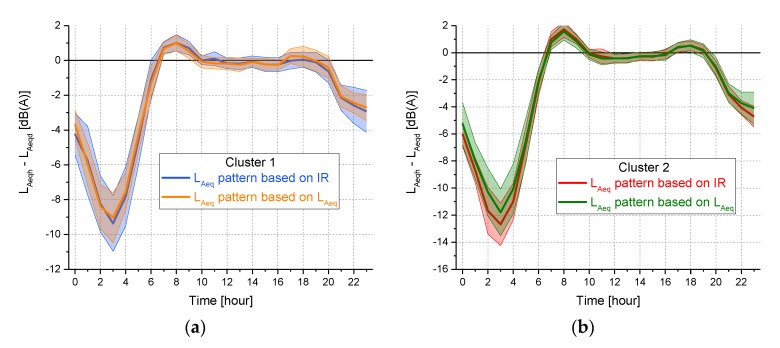
Comparison of the hourly *L_Aeq_* patterns corresponding to the two clusters (**a**, cluster 1 and **b**, cluster 2) obtained by the two classifications based on *IR* and *L_Aeq_*, respectively.

Despite the observed mismatch between the above two classifications, the difference between the hourly *L_Aeq_* patterns corresponding to the clusters obtained by the two classifications was not statistically significant at 95% confidence level for any hourly interval, even in the night period, as shown in Figure 14 where the hourly *L_Aeq_* median values ± the median absolute deviation (MAD) are reported. However, it has to be pointed out that the two classifications have different aims: the one based on *L_Aeq_* pattern is mainly focused on noise mapping, according to the standards issued by the European Directive 2002/49/EC [19], whereas that based on *IR* pattern could be aimed at discriminating the sites according to the potential annoyance their sonic environment might evoke. Thus, these two approaches are not alternative with one another but shall be considered complementary. Furthermore, both the classifications are rather different from the categorization based on the type of road, as established by the Italian legislation, which defines the noise limits as a function of the road category. Thus, this approach did not seem appropriate for an effective protection against road traffic noise pollution.

## 5. Conclusions

The intermittency ratio *IR* metric was applied to a database of road traffic noise, without being attended, monitored for 24 h in 90 sites in the city of Milan. The reference measurement time T was set at 1 h and the obtained *IR* values were processed by clustering methods. Two clusters were determined, providing hourly *IR* temporal patterns enabling us to classify the urban sites on the basis of the observed noise events, which, potentially, increase the annoyance. A “non-acoustic” parameter *x*, determined by combination of the traffic flow rate in three hourly intervals, was allowed to associate each site with the cluster membership. Furthermore, binomial logistic regression was applied to develop a model to predict the cluster membership on the basis of the *IR* time patterns. The performance of the model, determined comparing the predicted classification of the test data subset with that obtained by the cluster analysis, was satisfactory.

However, the *IR* values calculated from the noise data provided by the road traffic noise monitoring network in Milan, mainly used for a noise mapping update, had some drawbacks due to some factors, like different distances microphone-longitudinal axis of the road and microphone position close to the road and not where the residents live. The reference measurement time *T* chosen, equal to 1 h, had also affected the *IR* values. In addition, the results of clustering and classification model were strongly dependent on the local situation and could not be generalized to other contexts. However, the study showed that data collected for noise monitoring and mapping purposes could be processed to evaluate the occurrences of noise events produced by a vehicle pass-by. Besides the above limitations, the described methodology could be fruitfully applied on road traffic noise data in other cities and some general considerations could be drawn. In particular, *IR* could be a supplementary metric accompanying *L_Aeq_*, as the former describes SPL short-term temporal variation and the latter measures the energy content of the noise exposure. Indeed, *IR* could explain deviations of highly annoyed people percentage from that estimated by the classical exposure–response curves that only rely on *L_Aeq_* [4], like those in [20].

Furthermore, the two classifications based on *IR* and *L_Aeq_* hourly time patterns are rather different from that based on the type of road, as established by the Italian legislation, which defines the noise limits as function of the road category. Thus, this approach does not seem appropriate for an effective protection against road traffic noise pollution.

Further steps of this research are already planned and they include the statistics of errors in the estimate of *IR* values derived by the application of the above time patterns, as well as the potential of *IR* to detect correctly the noise events produced by road traffic, identified by an automatic recognition algorithm already developed within the DYNAMAP project [21,22].

## Figures and Tables

**Table 1:** Distribution of the 90 sites included in the road traffic noise monitoring.

Road Type	N. of Sites	N. of 24-h Time Series	N. of Lanes for Each Direction	Average Distance Microphone-Roadside [m]
A	2	15	3 (50.0%)	18.5
			4 (50.0%)	12.5
D	7	18	1 (28.6%)	5.5
			2 (14.3%)	7.0
			3 (42.8%)	7.0
			4 (14.3%)	4.5
E	29	83	1 (32.1%)	6.8
			2 (35.7%)	5.8
			3 (28.6%)	4.4
			4 (3.6%)	8.5
F	52	135	1 one way (30.0%)	9.0
			1 (56.0%)	10.5
			2 one way (2.0%)	6.5
			2 (12.0%)	13.0
Total	90	251		

**Table 2:** Distribution of the 90 sites across the two clusters and type of road for the classification based on *IR* hourly time patterns.

Cluster		1	2
**N. of sites (%)**		34 (37.8)	56 (62.2)
**Road Type**	A	2 (100)	0 (0)
	D	4 (57.1)	3 (42.9)
	E	16 (55.2)	13 (44.8)
	F	12 (23.1)	40 (76.9)

**Table 3:** Classification performance of the logistic model.

Parameter	Value
Sensitivity	0.71
Specificity	0.88
Detection rate	0.21
Balanced accuracy	0.80
